# The Internet for weight control in an obese sample: results of a randomised controlled trial

**DOI:** 10.1186/1472-6963-7-206

**Published:** 2007-12-19

**Authors:** Áine McConnon, Sara FL Kirk, Jennie E Cockroft, Emma L Harvey, Darren C Greenwood, James D Thomas, Joan K Ransley, Laura Bojke

**Affiliations:** 1 Food, Consumer Behaviour and Health Research Centre, University of Surrey, Guildford, Surrey, UK; 2 Nutritional Epidemiology Group, Centre for Epidemiology and Biostatistics, University of Leeds, Leeds, UK; 3 Biostatistics Unit, Centre for Epidemiology and Biostatistics, University of Leeds, Leeds, UK; 4 Centre for Health Economics, University of York, Heslington, York, UK

## Abstract

**Background:**

Rising levels of obesity coupled with the limited success of currently available weight control methods highlight the need for investigation of novel approaches to obesity treatment. This study aims to determine the effectiveness and cost-effectiveness of an Internet-based resource for obesity management.

**Methods:**

A randomised controlled trial conducted in a community setting, where obese volunteers (n = 221) were randomly assigned to Internet group (n = 111) or usual care group (n = 110). Objective measures of weight and height were obtained. Questionnaires were used to collect dietary, lifestyle, physical activity and quality of life data. Data were collected at baseline, six months and 12 months.

**Results:**

Data were collected on 54 (49%) participants in the Internet group and 77 (70%) participants in the usual care group at 12 months. Based on analysis conducted on all available data, the Internet group lost 1.3 kg, compared with 1.9 kg weight loss in the usual care group at 12 months, a non-significant difference (difference = 0.6 kg; 95% CI: -1.4 to 2.5, p = 0.56). No significant differences in change in secondary outcome measures between the two groups at six or 12 months were revealed. Total costs per person per year were higher in the Internet group than the usual care group (£992.40 compared to £276.12), primarily due to the fixed costs associated with setting up the website, and QALYs were similar (0.78 and 0.77) for both groups.

**Conclusion:**

This trial failed to show any additional benefit of this website in terms of weight loss or secondary outcome measures compared with usual care. High attrition and low compliance limits the results of this research. The results suggest that the Internet-based weight control resource was not a cost-effective tool for weight loss in the obese sample studied.

**Trail Registration:**

ISRCTN 58621669

## Background

With 22.7% of men and 23.8% of women in England classified as obese, obesity is now widely recognised as a major public health issue [1]. Successful weight loss is impeded by a multitude of factors, including limited reach of currently available treatments [2], lack of social support [3], poor compliance [4], changing environmental factors such as ready availability of cheap energy dense foods and increased existence of labour-saving devices [5] and, for many, dissatisfaction with the structured nature of many treatments [6]. These shortcomings highlight the need to investigate and evaluate new approaches to obesity treatment. New approaches should also aim to reduce the burden on those participating and delivering the intervention, thereby promoting a more flexible approach to weight management [7], factors that could be addressed using new technologies such as the Internet.

In July 2003 it was estimated that 47% of households in the UK had access to the Internet at home. In 2005 this figure had risen to 55% [8]. The rapid increase in access to the Internet and use of the Internet as a health resource has made it a viable tool for health care interventions [9], offering anonymous, self-administered care that is available 24 hours a day, seven days a week, with little time or travel costs to the individual. A wide array of Internet-based weight loss resources exist [10]. However, despite the huge presence of such information on the Internet, little is known about their use or effectiveness. A study by Womble *et al *recently reported that a commercial Internet-based weight loss program was less effective than a standardised, structured behavioural weight loss manual in terms of weight loss [11]. Other studies have demonstrated successful weight loss and weight loss maintenance via Internet and e-mail programmes [9,12,13].

The aim of this project was to develop an Internet-based weight-control package, requiring minimal professional input, and to test this package against usual care for obesity treatment in a community setting, through a pragmatic randomised controlled trial. We also sought to estimate the cost-effectiveness of the alternative programs for weight management. This trial represents the first attempt to assess the effectiveness and cost-effectiveness of an Internet-based weight control package against usual care for the management of obesity.

## Methods

### Participants

Participants were recruited from GP practices in Leeds, UK, following ethical approval from Leeds (West) Research Ethics Committee in August 2002. Posters and flyers were placed in patient waiting areas, advertising the study and asking interested potential participants to call the study centre or to inform their GP or practice nurse of their interest. Potential participants were screened by telephone for eligibility. Eligibility criteria included: individuals with a body mass index (BMI) of 30 or more, aged 18–65 years (due to body composition changes over the age of 65 years), able to access the Internet at least once per week and able to read and write in English (for the purposes of accessing the website and completing questionnaires).

On confirmation of informed written participant and GP consent, a baseline appointment was scheduled for each eligible participant, where height and weight were measured by the researcher and a baseline questionnaire completed, before each participant was randomly allocated into either the Internet group (n = 111) or the usual care group (n = 110). Weight (in light clothing) was measured to the nearest 0.1 kg using Marsden portable weighing scales. Height (without shoes) was measured using a portable height measure to the nearest 0.1 cm. Recruitment into the trial took place from May to November 2003. Participants were followed-up six months and 12 months after randomisation, when height and weight measurements were repeated by the researcher, along with a follow-up questionnaire administered by post. Baseline and follow-up appointments were conducted at the participants' GP practice.

All non-responders were followed up following a strict protocol by post and telephone. Three attempts were made to contact each participant not responding to the initial mailing at follow-up by telephone, following this a second questionnaire was mailed to participants asking them to complete the questionnaire even if they did not wish to attend a follow up visit.

### The intervention

Current research evidence supports a lifestyle approach to treating obesity, offering a combination of dietary advice, physical activity advice and behaviour therapy [14-17]. Based on these guidelines and clinical evidence, the intervention website was developed to reflect these factors. The website provided advice, tools and information to support behaviour change in terms of dietary and physical activity patterns. It was designed to enable patients to manage their own care and to vary the frequency of use according to their own needs. The website also offered personalised advice to participants, which, in the context of this trial, involved targeting the information provided to an individual, based on their responses to a series of online questions regarding eating and activity habits and current weight status. This enabled specific motivational statements to be generated to participants whenever they visited the website. Motivational statements were generated based on participants self report of progress in terms of reaching their personal behaviour change goals. In addition, details of progress in terms of self-reported weight loss were stored on the website, accessible only to the individual concerned. Automatic generic e-mails were generated if participants did not visit the website regularly to encourage them to visit more often. The website and questionnaires were piloted in a sample of overweight University staff. The results of this pilot were used to inform the final version of the website.

The trial aimed to compare the additional effect of the website against usual care available within the UK. Participants randomised into the Internet group were given a demonstration of the website and its services, along with a username and password to access the website and were asked to log on to the website at least once a week over the trial period. Participants randomised to the usual care group were advised to continue with their usual approach to weight loss and were given a small amount of printed information at baseline, reflecting the type of information available within primary care.

### Outcomes

The primary outcome was the ability of the Internet package to promote change in weight and BMI over six and 12 months compared with usual care. Secondary outcomes were the ability of the Internet package to promote change in reported lifestyle behaviours compared with usual care, along with differences in quality of life. Lifestyle and dietary habits were assessed with a questionnaire previously used in the UK Women's Cohort Study [18]. This questionnaire obtained information on methods of cooking, portion size and frequency of consumption of various foods and participants' 'dieting' practices. Physical activity level was assessed using the Baecke physical activity questionnaire which measures work, leisure and sports activity providing a comprehensive evaluation of habitual physical activity [19]. Quality of life was assessed using the EuroQol questionnaire, a short, self administered questionnaire which was also used in the cost effectiveness analysis [20]. A brief series of questions were used to assess participants' confidence in their ability to make positive lifestyle changes on a scale of one to seven (where one indicates not at all confident and seven indicates very confident). These measures were combined in one questionnaire, which participants completed at baseline, six months and 12 months. Additional questions were added at six and 12 months for the purpose of the cost-effectiveness analysis of the programme. Participants in the Internet group also completed an additional section on their use and views of the website at six and 12 months.

### Sample size

A sample size of 180 participants was required to detect a difference of 5 kg weight loss (approximately 5% of body weight) or less than 2.5 kg/m^2 ^in BMI between the two groups with 80% power, assuming a two-sample t-test, 5% significance levels, a standard deviation for weight of 12 kg and for BMI of 5.5 kg/m^2^. An additional 22% of participants were recruited to take account of any loss to follow-up, giving a recruitment target figure of 220.

### Randomisation

A computer-generated randomisation procedure was employed, using the software package 'minim' [21]. Participants were allocated to groups by the programme according to the minimisation criteria, i.e. balanced for gender (male/female), age group (18–34, 35–49, 50+) and BMI category (30–33.9, 34–37.9, 38+). Due to the pragmatic nature of the trial and the intervention being evaluated, it was not possible to blind either the participants or researchers to the group assignment.

### Statistical analysis

Analyses were performed using Statistical Package for the Social Sciences (SPSS for Windows, version 11.5; SPSS, Chicago, IL). Independent sample t-tests (or non-parametric tests where appropriate) and chi-squared tests were used to investigate differences in baseline characteristics and response rates between the two groups. Analysis of covariance (ANCOVA) on weight at 12 months was used to investigate the difference between the two groups. This adjusted for any imbalance in age, sex, baseline weight, baseline physical activity score or baseline confidence score introduced by losses to follow-up. Changes in secondary measures were investigated using ANCOVA adjusting for possible baseline imbalances as before. Primary analyses were conducted based on all available data. Analyses using LOCF and BOCF were performed to assess the robustness of the primary analysis for the effect of losses to follow up and missing data.

### Cost-effectiveness analysis

The cost-effectiveness analysis was undertaken in Stata SE 8.2. Quality adjusted life years (QALYs), calculated using utilities collected by the EuroQoL (EQ-5D), were compared with total costs estimated using a societal perspective. Costs were estimated from a variety of sources, including the Personal Social Services Research Unit (PSSRU) for visits to the GP and practice nurse [22]. Adjusted (for characteristics of patients that differed between the group) estimates of mean cost and QALYs were obtained by a multi-level difference-in-difference econometric model [23]. The incremental cost-effectiveness of the two alternative methods of weight loss support based on mean differential costs and QALYs was then established. Finally the probability that Internet-based support is cost-effective according to a range of alternative threshold values, which the health care system may be willing to pay for an additional QALY, was calculated [24]. This is then plotted as a cost-effectiveness acceptability curves (CEAC) [24]. The CEAC shows the proportion of simulations (produced by the econometric model) in which web-based support (and conversely traditional weight loss) is the more cost-effective across a range of alternative threshold values, which the health care system may be willing to pay for an additional QALY.

## Results

### Baseline characteristics

The sample was predominantly white (95%), female (77%), with a mean (sd) age of 45.8 (10.6). The mean (sd) weight of the sample was 98.4 kg (17.4) with a median (IQR) BMI of 34.4 (31.9–38.7). Preliminary analysis showed no significant differences between the two groups at baseline (Table 1).

**Table 1:** Subject characteristics at baseline

	Internet group (n = 111)	Usual Care group (n = 110)
**Weight**, mean (sd), kg	98.9 (17.7)	97.9 (17.1)
**Body Mass Index (BMI)**, median (IQR), kg/m^2^	34.5 (31.8–38.5)	34.4 (31.9–38.9)
**Quality of Life **score, median (IQR)	70 (55–80)	65 (50–80)
**Physical activity **score, mean (sd)	6.8 (0.98)	6.7 (1.3)

### Response rates

**Figure 1:**
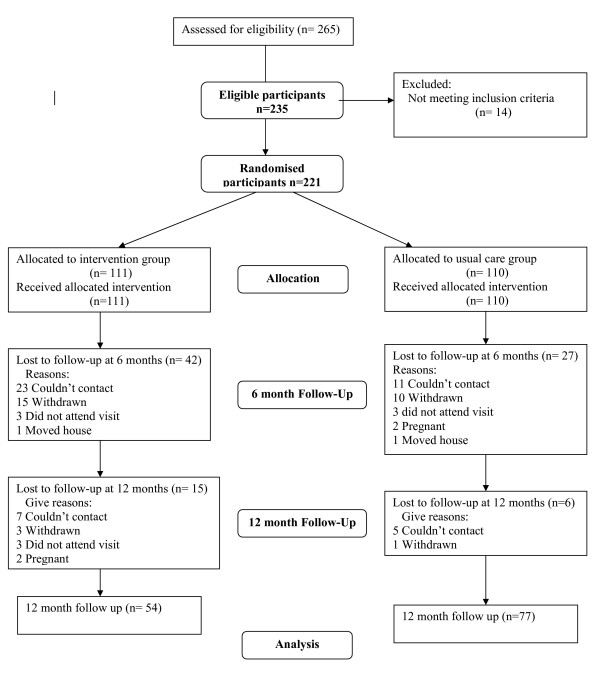
**Participant Flow**. This chart provides a graphical illustration of the flow of participants through the trial over the trial period.

Measurements were obtained for 69% (n = 152) of the sample at 6 months and for 59% (n = 131) at 12 months, equating to an attrition rate of 31% at six months and 41% at 12 months. Response rate in the group Internet was significantly lower than the usual care group at 12 months (49% versus 70%; p = 0.001). Participant flow through the trial is given in Figure 1.

Table 2 presents the baseline characteristics for responders only by treatment group. No differences in baseline characteristics of responders by treatment group were revealed except for age. Responders in the usual care group were significantly younger than responders in the Internet group (p = 0.003).

**Table 2:** Comparison of baseline characteristics of responders by treatment group

	Internet (n = 54)	Usual Care (n = 77)	Confidence Interval, p value.
**Weight**, kg, mean	97.5	94.9	(-8.3, 3.1) p = 0.24
**BMI**, median	34.35	34.4	p = 0.6
**Confidence score**, mean	4.2	4.1	(-0.37, 0.5), p = 0.07
**Physical activity score**, mean	6.8	6.7	(-0.3, 0.4) p = 0.23
**Quality of Life score**, median	70	61.5	p = 0.2
**Age**, years, mean	48.1	47.4	(-2.6, 4) p = 0.003*

*p < 0.05

Baseline characteristics of participants with a complete data set (responders) and non-responders are presented in Table 3. Responders at 12 months were significantly older (difference = 6.2 years; CI = -9.5 to -3, p < 0.001) and more confident (difference = 0.48; CI = 0.14 to 0.82, p = 0.006) than non-responders. The mean baseline weight for responders was 96.4 kg compared with a mean weight of 101.5 kg for non-responders. This difference was borderline significant (difference = 5.1 kg, CI = -0.3 to 10.3, p = 0.06). No gender differences in response were identified.

**Table 3:** Comparison of baseline characteristics of responders versus non-responders

	Responders	Non-responders	Confidence Interval, p value
**Weight**, kg, mean	96.4	101.5	(-0.25, 10.3), p = 0.06
**BMI**, median	34.4	37.2	p = 0.15
**Confidence score**, mean	4.1	4.6	(0.14, 0.82), p = 0.006*
**Physical activity score**, mean	6.75	6.58	(-0.5, 0.2) p = 0.34
**Quality of Life score**, median	63	63.67	p = 0.96
**Age**, years, mean	47.7	41.5	(-9.5, -3.0), p =< 0.001**
**Gender**, Male/Female %	21%/79%	22%/78%	p = 0.95

*p < 0.05, **p < 0.001

### Change in weight and BMI

**Figure 2:**
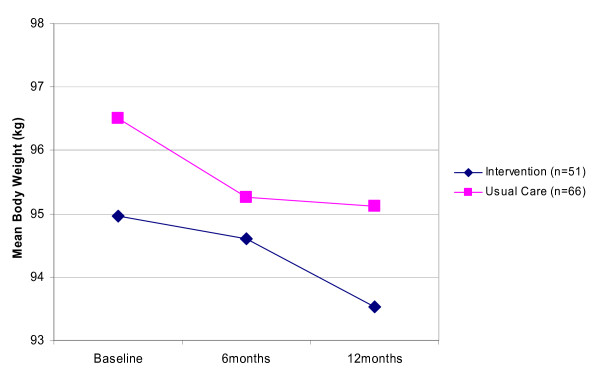
Change in body weight by trial group (based on 117 participants who completed all three assessments). Differences between the groups at six and 12 months were non significant.

Change in BMI between the two groups at 12 months was non-significant, with a mean difference of 0.3 kg/m^2 ^(CI = -0.5 to 1, p = 0.4), ranging from -5.9 kg/m^2 ^to +3.8 kg/m^2 ^for the Internet group and -8.1 kg/m^2 ^to +3.5 kg/m^2 ^for the usual care group at 12 months. Both groups lost a significant amount of weight over time, but the difference in change between the groups at 12 months was non-significant. ANCOVA using weight at 12 months as the dependent variable, adjusting for age, sex, baseline weight, baseline physical activity score and baseline confidence score revealed that the Internet group were 0.6 kg heavier (95% CI: -1.4 to 2.5, p = 0.56) than the usual care group after 12 months (Figure 2). Similar results were produced from BOCF data (Internet group 0.8 kg heavier (95% CI: -0.4 to 1.9, p = 0.2)) and LOCF data (Internet group 0.5 kg heavier (95% CI: -0.8 to 1.8, p = 0.4)), demonstrating the robustness of the results to alternative assumptions. We were also interested in the loss of 5% body weight, as this is associated with significant improvements in health [25]. Investigating weight loss as a percentage of the baseline weight, 22% of Internet responders lost 5% or more of their baseline weight by 12 months, with 18% of the usual care group losing at least this amount.

### Secondary measures

A significant reduction in self-rated physical activity score over the intervention period was identified (p < 0.005) with a mean reduction of 0.34 in the sample, the difference in change between the two groups over the 12 months was non-significant (p = 0.6). Quality of life was shown to increase significantly over the trial (p = 0.03), however no between group differences were established (p = 0.8). No significant changes in dietary habits or confidence scores were detected over the 12 months.

### Use of the website by Internet group participants

Fifty-nine participants (53%) reported using the website at six months with 32 (29%) of these still using the website at 12 months and 52 participants (47%) indicating that they never used the website. The mean (sd) number of logons over the trial was 15.8 (15.2), this ranged from a minimum of one logon to a maximum of 77 logons. The data failed to reveal any correlation between the number of logons and weight loss (p = 0.16). Only 26% of the Internet group respondents at six months reported using the Internet daily for general use and no relationship between Internet use and number of logons to the website over the 12 months was established (CI = -10.1 to 12.9, p = 0.82). Despite high attrition and low utilisation, of those who had reported use of the website at six months 39 (63%) rated it easy or very easy to use at six months, and 49 (78%) rated it as clear or very clear. With 28 (85%) and 25 (76%) of those respondents reporting use of the website in the second six months of the trial rating it as easy or very easy and clear or very clear at 12 months.

As was the nature of this pragmatic trial participants were free to engage in the use of other weight loss resources. Participants' use of other weight control resources was recorded and no differences in use between the two groups were evident at six or 12 months.

### Cost-effectiveness results

The cost-effectiveness analysis showed that total costs were higher in the Internet group than the usual care group (£992.40 compared to £276.12). This difference was almost entirely due to the fixed cost of developing the website package. When this fixed cost was excluded total costs were actually lower in the Internet group. QALYs were similar (0.79 compared to 0.77). Results are shown in Table 4.

**Table 4:** Expected yearly outcomes from the econometric model

	**Internet**	**Usual care**	**Incremental difference**
**QALY**	0.79	0.77	+0.02
**Cost (excluding fixed cost)**	221.09	276.13	-£55.04
**Total cost**	992.41	276.13	+£716.28

**Figure 3:**
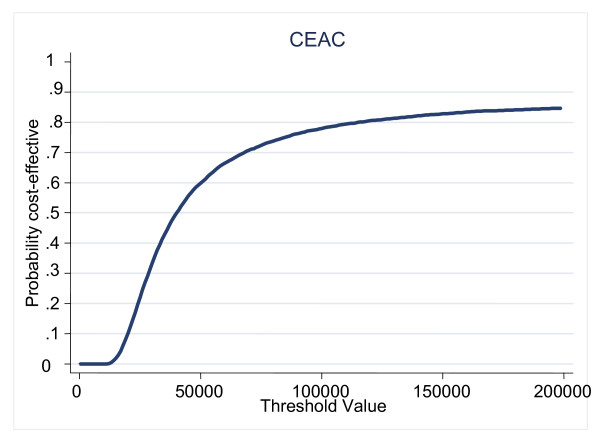
Cost-effectiveness acceptability curve for web-based support.

In terms of incremental cost effectiveness [26] the incremental ratio is £39,248 (£716.28/0.01825). Thus a decision maker would have to be willing to pay £39,248 per QALY to choose the Internet program over the usual care approach. AS shown in the cost-effectiveness acceptability curve (Figure 3) the decision about which is the most cost-effective strategy is uncertain. At willingness to pay values of £20,000–£30,000 per QALY, it is unlikely that Internet-based support would be regarded as cost-effective (probability it is cost-effective is less than 0.5). As the service becomes more widely available, fixed costs will be spread over a greater number of individuals, thus total costs will reduce. This is likely to produce a much more favourable cost per QALY.

## Discussion

This trial aimed to test the effectiveness of a website designed for weight control, based on a self-help approach, in comparison to the standard weight control options available in a 'real life' setting and consequently to assess the feasibility of the resource as an adjunct for weight management. Although a significant weight loss was detected over the trial period, no difference in weight change between the two groups was detected. Weight losses reported in previous research studies using the Internet have not been as good as that reported for standard behavioural face to face therapy [27]. In a recent study by Womble *et al *comparing a commercially available online weight loss resource with a structured weight loss manual, the Internet group lost only 1.1% (sd = 4.0%) of their baseline weight by 12 months compared with a mean (sd) weight loss of 4.0 (5.1%) in the manual group [11]. The lack of intervention effect over and above what happened over time in both groups may be explained by the poor engagement with the intervention tool. With just over half the participants using the website at six months, only one third were using it at 12 months. Womble *et al *also reported minimal use of the intervention website [11]. Recent research has shown that participants are more likely to attend group sessions than use Internet chat rooms in a weight control trial [28]. However, despite high attrition and low utilisation, data collected as part of this research indicated that the majority of respondents (>60%) felt the website was clear/very clear and easy/very easy to use both at six and 12 months. Although the reasons for the decline in use of the resource are not known it is likely that the lack of traffic on the website led to a reduction in the activity on the website and thus reduced the ability of the resource to engage participants. It is expected that a larger sample size would have increased the 'traffic' on the website and consequently increased the social support element of the intervention. It is also possible that participant expectations were not met, thus leading to a decline in use of the intervention. It has been suggested that unmet goals and expectations can result in individuals abandoning their attempts to achieve weight loss [29]. An association between personalised feedback and success in weight loss has been established previously [30]. Increased personalised feedback could be one of the keys to increasing compliance and success of the Internet-based weight control resources. To increase compliance in web-based interventions, further investigation of the role of mechanisms known to increase compliance in traditional face-to-face interventions such as tailored feedback and professional and peer support of varying degrees should be investigated.

Attrition in randomised trials of weight loss is recognised as a major issue in evaluating the effectiveness of weight control treatments [31]. An average attrition rate of 21% is typical in lifestyle interventions for weight loss [27], in a recent review of long term weight loss studies in obese adults losses to follow-up typically between 30–60% were reported [32]. An overall attrition rate of 40% in this trial was higher than anticipated. Attrition rates as high as 34% have been reported in weight loss trials using the Internet [11]. One possible reason such high attrition may be limited weight loss. Other Internet intervention studies achieving greater weight losses have demonstrated much lower attrition rates [9,12]. High attrition rates in obesity research limit the ability of trials to effectively evaluate treatments and present a major barrier in weight loss research. The implications of the high attrition rate on the results of this trial are difficult to predict, however the analyses demonstrate that the results are not sensitive to the different assumptions for missing data providing confidence in the data. As recommended by Womble *et al *future research should aim to evaluate such Internet-based weight control websites in the manner in which the public uses them, with much larger sample sizes in collaboration with industry as this is likely to make the intervention more engaging and thus induce compliance [11].

In previous studies the 'self care' approach has been shown to offer promising strategies for weight loss [33]. This resource offered participants the freedom to access the website at their convenience, according to their perceived need. Womble *et al *suggested lack of structure limited the potential benefits of eDiets.com [11] and this may be true for this resource also. The website aimed to offer a low maintenance weight loss tool with limited professional support. It is hypothesised that this lack of regular professional support may have reduced the potential success of this website and that, in keeping with other studies, a balance between Internet and personal contact may offer a more optimal approach for weight management. Also it has been suggested that such a self help approach may be more useful as an adjunct to more intensive treatment [33]. A recent study by Wing *et al *suggested the use of the Internet for prevention of weight regain but that more intensive face-to-face intervention may be required if weight is regained [34].

One potential barrier to the use of this resource was that the majority of this population did not use the Internet on a daily basis. With only a quarter of responders at six months reporting use of the Internet on a daily basis, targeting this resource to individuals who use the Internet more regularly and who have greater confidence in their ability to use it may be more appropriate. In addition future research should aim to investigate the potential role of such resources in different at risk sub-groups of the population including low-income groups and ethnic minorities.

In terms of cost-effectiveness, the Internet-based support in this trial does not seem to fall within accepted standards for the incremental cost-effectiveness ratio. This is mainly due to the high fixed cost of setting up and running the program (£771 per participant in the Internet group), which made it substantially more costly than the usual care group to set up. However, as the intervention is Internet-based, its use by a larger pool of participants could improve cost-effectiveness.

## Conclusion

In conclusion, this trial failed to demonstrate any additional benefits in terms of weight management from the Internet-based tool. Limited use of the Internet in general in this population may have reduced the ability of this website to produce a significant weight loss. The high attrition rate and limited use of the website limits how representative these findings are. Although lack of engagement in Internet-based resources for weight loss presents a major obstacle in this field of research, use of the Internet as a tool in obesity management should not be dismissed as it still has the potential to offer wide reach for public health interventions. Consumer preferences and views of 'online weight control' should be considered in future research investigating effectiveness of Internet-based tools for the management of obesity in an effort to improve compliance with such interventions.

## Competing interests

The author(s) declare that they have no competing interests.

## Authors' contributions

SK was the Principal Investigator, who conceived of the idea and obtained funding for the research. AM was the research manager and was responsible for data collection, data entry, analysis and interpretation and drafting the publication and assisted in developing research protocols and questionnaires. EH, JR, JC provided advice and guidance on the study design and conduct of the RCT. JT was the database manager and web master. DG advised on the statistics and LB conducted the economic analysis.

## Pre-publication history

The pre-publication history for this paper can be accessed here:


